# Splenosis mimicking an extramural duodenal mass: A case report

**DOI:** 10.3892/ol.2014.2609

**Published:** 2014-10-10

**Authors:** YILEI DENG, YANWEN JIN, FUYU LI, YONG ZHOU

**Affiliations:** Department of Biliary Surgery, West China Hospital, Sichuan University, Chengdu, Sichuan 610041, P.R. China

**Keywords:** splenosis, splenectomy, malignancy, mass, biopsy

## Abstract

Splenosis is a common disease, patients with splenosis are generally asymptomatic and therapy is not indicated. Splenosis is frequently observed in the abdomen and pelvic cavity and may mimic malignancy on imaging, often leading to unnecessary surgical intervention. The current study presents the case of a 55-year-old female patient, with a rare case of duodenal splenosis, who underwent unnecessary laparotomy due to a misdiagnosis of a malignant duodenal stromal tumor. Although splenosis was confirmed by intraoperative tissue biopsy, this mass was resected due to the lack of information with regard to this condition, an increased suspicion of progressive growth of the mass and chronic duodenal compression. The aim of this report is to raise the awareness of this entity in patients post-splenectomy, to avoid unnecessary surgery, particularly with an increased prevalence of patients with previous splenic trauma due to road traffic accidents. Therefore, the possibility of abdominal splenosis must be included in the differential diagnosis of patients with abdominal mass as the main clinical manifestation, where there is a history of splenic trauma or splenectomy and no other systemic symptoms. In the future noninvasive nuclear scintigraphy may serve as a suitable diagnostic approach for splenosis, thereby avoiding unnecessary laparotomies.

## Introduction

In 1939, Buchbinder and Lipkopf initially used the term ‘splenosis’ to describe the heterotopic autotransplantation of splenic tissues (1); following this, reports of such conditions are gradually increasing. Currently, splenosis is not considered to be a rare disease, and the incidence of splenosis in patients with spleen trauma or splenectomy may be ≤67% (2). However, splenosis is often diagnosed incidentally. Splenosis is commonly observed in the abdomen and pelvic cavity; it mimics malignancy on abdominal imaging, regardless of the signs and symptoms, and may also lead to unnecessary surgical interventions (3). The current study reports a case of duodenal splenosis located outside of the descending section of the duodenum. The patient underwent unnecessary laparotomy due to a significant diagnostic dilemma, as the possibility of a malignant tumor could not be eliminated. Written informed consent was obtained from the patient.

## Case report

**Figure 1:**
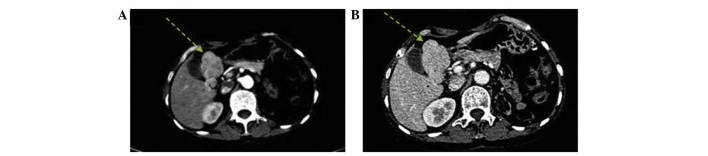
CT imaging of the abdomen. CT imaging in the (A) arterial and (B) portal phases revealed no spleen and a maximum diameter of 6.1×3.6 cm mass (arrows) arising from the duodenal bulb, mimicking a duodenal tumor.

A 55-year-old female was admitted to the Department of Biliary Surgery, West China Hospital (Chengdu, China) with a history of duodenal mass, which was identified following a routine physical examination in a local hospital. No systemic symptoms were observed and there was no history of malignancy or weight loss. The patient had a history of splenectomy following a traumatic spleen rupture due to a traffic accident 27 years previously. No history of drug and alcohol abuse was evident, the patient had a normal dietary history and had not previously visited the nomadic areas, where echinococcosis is prevalant. Physical examination and the initial laboratory tests at the West China Hospital revealed no abnormalities with the exception of the presence of a postoperative abdominal scar in the left upper quadrant. Abdominal ultrasound revealed no visual spleen due to the splenectomy, and a hilar mass measuring 3.9×2.6×4.9 cm with a rich blood supply. Subsequent contrast helical computed tomography (CT) imaging showed no spleen and a mass with a maximum diameter of 6.1×3.6 cm, mass arising from the duodenal bulb (Fig. 1), which was considered to be a malignant duodenal stromal tumor.

**Figure 2:**
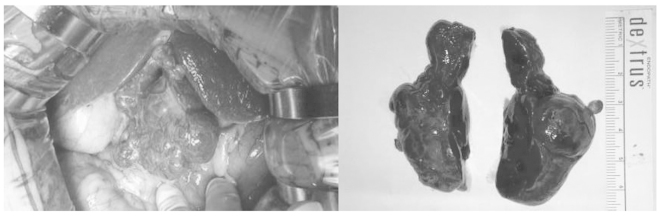
Intraoperative image showed a dark red multi-nodular soft tissue mass of 6.5×4.8×2.6 cm in size among the duodenal bulb, hilar and the gastric antrum, suggesting ectopic splenic tissue.

Following preoperative preparation and counselling with the patient and relatives, exploratory laparotomy was conducted. During surgery, a multi-nodular soft tissue mass with a dark red appearance, of 6.5×4.8×2.6 cm in size was observed around the duodenal bulb, hilar and the gastric antrum, with a blood supply from the duodenal wall (Fig. 2). The mass was predicted to be spleen tissue, confirmed by intraoperative tissue biopsy, however, the mass was resected to avoid any future problems that may arise. Following this, the patient was diagnosed with post-splenectomy duodenal splenosis, and was discharged on postoperative day 8 without any complications. During hospitalization, the platelet count increased from a preoperative level of 165×10^9^/l (reference range, 100×10^9^/l–300×10^9^/l) to 314×10^9^/l on the day of discharge. During the six month follow-up, the patient was asymptomatic and monthly ultrasonography revealed no abdominal abnormalities, however, the platelet count continued to rise to a maximum of 425×10^9^/l in the first month following surgery, and thereafter, a progressive decline was observed and subsequently, platelet levels remained within the normal range.

## Discussion

The majority of cases of splenosis are the result of post-traumatic splenectomy (4). Following splenic rupture, damaged splenic pulp seeds in the adjacent cavities, and grows using the blood supply from adjacent blood vessels (5). Duodenal splenosis is rare, and splenosis was not predicted in this patient prior to surgery. A preliminary diagnosis of splenosis may be determined with caution. A detailed medical history, including any instance of post-traumatic splenectomy, and thorough physical examination is essential, however further tests are required to confirm the diagnosis. X-ray, ultrasound, CT and standard magnetic resonance imaging are of limited value in the diagnosis of abdominal splenosis (3,6). These imaging findings are nonspecific in this entity, indicating the size, shape, number and location of the masses, but cannot distinguish splenosis nodules from numerous conditions such as metastatic disease; this occurred in the present case. Technetium-99 m heat-damaged erythrocytes (RBC) or Indium 111-labeled platelets scintigraphy, have been accepted by an increasing number of clinicians as a noninvasive nuclear scintigraphy for the diagnosis of splenosis, due to the ability of spleen tissue to absorb radio-labeled, damaged red blood cells (7,8). Notably, RBC scintigraphy has been demonstrated to exhibit increased sensitivity in early splenosis, functional hyposplenism and poor splenic uptake (9,10). However, despite these advanced imaging techniques, a pathological diagnosis of splenosis is usually required, predominantly due to the possibility of malignancy or to preoperative diagnostic uncertainty. Therefore, in the present case, a laparoscopic approach was adopted, providing a minimally invasive method to directly visualize the suspected mass, allowing the biopsy and resection, if required (11).

This was the first case of splenosis in the Department of Biliary Surgery, West China Hospital. Due to insufficient information with regard to this condition and increased suspicion with regard to the progressive growth of the mass, leading to duodenal compression, resection appeared to be urgent. Splenosis is a benign condition and usually asymptomatic, therefore, the removal of an asymptomatic splenosis mass is not required. Additionally, it has been reported that splenic implants may exert a protective immune response against bacterial infections in asplenic patients, however, this effect is limited (12). In the present case, the gradual rise in platelet count during the month following surgery was similar to the change in platelet levels observed in other splenectomized patients, where platelet levels increased before returning to normal levels, suggesting a functional reticuloendothelial system within the splenosis (13).

Splenosis is not a rare disease. In patients with a history of splenic trauma or splenectomy and an abdominal mass as the predominant clinical manifestation, particularly in the absence of systemic symptoms, abdominal splenosis must be included in the differential diagnosis. Once considered, combined with a comprehensive history, noninvasive nuclear scintigraphy may serve as a suitable diagnostic approach for splenosis, thereby avoiding unnecessary laparotomy. In cases where a pathological diagnosis of splenosis is required due to concern of malignancy or preoperative diagnostic uncertainty, a laparoscopy as a minimally invasive technique, may be utilized. Following confirmation, the removal of splenosis nodules is not required in asymptomatic cases, as splenosis is harmless and may exert beneficial effects in asplenic patients.
